# DEMENTIA CAREGIVER PERCEPTIONS OF TELE-DEMENTIA CARE FOR VETERANS DURING THE COVID-19 PANDEMIC

**DOI:** 10.1093/geroni/igac059.2096

**Published:** 2022-12-20

**Authors:** Sowmya Iyer, Victoria Ngo, Marika Humber, Marisa Brodrick, Christine Gould, Ranak Trivedi

**Affiliations:** VA Palo Alto Healthcare System, Palo Alto, California, United States; VA Palo Alto Health Care System, Menlo Park, California, United States; VA Palo Alto Health Care System, Palo Alto, California, United States; VA Palo Alto Healthcare Syste, Palo Alto, California, United States; VA Palo Alto, Palo Alto, California, United States; Stanford University, Palo Alto, California, United States

## Abstract

The estimated 5 million persons living with dementia in the United States have been greatly impacted by the medical and psychosocial impacts of the COVID-19 pandemic, respite program closures, social isolation, and Veterans seen within the Veterans Health Administration system are particularly vulnerable. Telemedicine provides needed specialty dementia care to these patients with complex needs in their homes, and its uptake has increased during the pandemic. This qualitative, observational study explored informal caregivers’ perceptions of tele-dementia care for Veterans seen at 2 sites, Palo Alto and Cleveland, via semi-structured interviews. Twenty-five caregivers (Mean age = 67y, SD=12y, 88% women) were interviewed over telephone following a tele-dementia visit. Themes that emerged from the interviews were that tele-dementia visits: (1) saved caregivers 2.6h±1.5h (Range: 0.5 to 6h) of travel time, (2) required limited preparation compared to in-person visits, (3) mitigated COVID-19 risk and avoided needs for masking and social distancing, (4) avoided behavioral challenges during appointments, and (5) allowed participation from home with minimal disruption of routine. Caregivers described significant physical challenges that made leaving the home for appointments difficult including balance issues, incontinence, and difficulties getting into vehicle. Caregivers plan to continue using tele-dementia services beyond the pandemic due to the convenience. Taken together, these findings indicate that caregivers find tele-dementia care convenient, comfortable, helpful, and timesaving and highly satisfactory. A combination of both in-person and virtual visits would be an ideal future state. This study illustrates how caregivers experience virtual visits for dementia care and will shape future intervention design.

